# The Effectiveness of Disaster Education for Undergraduate Nursing Students’ Knowledge, Willingness, and Perceived Ability: An Evaluation Study

**DOI:** 10.3390/ijerph181910545

**Published:** 2021-10-08

**Authors:** Maria Shuk Yu Hung, Stanley Kam Ki Lam, Meyrick Chum Ming Chow, Winnie Wing Man Ng, Oi Kiu Pau

**Affiliations:** 1 School of Nursing, Tung Wah College, Hong Kong, China; meyrickchow@twc.edu.hk (M.C.M.C.); okpau@twc.edu.hk (O.K.P.); 2 The Nethersole School of Nursing, The Chinese University of Hong Kong, Hong Kong, China; stanleylam@cuhk.edu.hk; 3 Division of Science, Engineering and Health Studies, College of Professional and Continuing Education, The Hong Kong Polytechnic University, Hong Kong, China; winnie.ng@cpce-polyu.edu.hk

**Keywords:** knowledge, willingness, ability, disaster, training, nursing students

## Abstract

As future healthcare professionals, nursing students should possess the appropriate knowledge, skills, and positive attitude to respond to public health emergencies or disasters worldwide. This study evaluated the effectiveness of a disaster management training course at improving Hong Kong nursing students’ disaster knowledge, willingness, and perceived ability. A mixed-method design using a single group with pre- and post-intervention comparisons followed by qualitative focus group interviews, was conducted. A 45-h disaster management training course with theoretical and practical inputs was conducted. A total of 157 students participated in and completed the pre- and post-intervention questionnaires. Positive significant results in disaster knowledge (t(156) = −8.12, *p* < 0.01, d = −0.84) and perceived ability (t(156) = −7.95, *p* < 0.01, d = −0.72) were found, but no substantial change in willingness to respond to disasters was observed. The participants expressed various concerns regarding their willingness to respond, which can be summarized and grouped as (1) personal risk perceptions, (2) contextual factors of the disaster events, and (3) organizational support. Incorporating disaster training into the tertiary education curricula for basic nursing professionals’ training could be a long-term strategy to prepare and expand the competent workforce for future disasters. Government or healthcare organizations are recommended to provide strategies and adequate support to alleviate nursing professionals’ concerns and enhance their willingness.

## 1. Introduction

The increasing trend of unpredictable natural or human-caused disasters worldwide has provoked extreme human health, economic, and environmental impacts in recent years. Healthcare professionals’ capacity and obligation to provide services during public health emergencies or disasters are indispensable elements of disaster management. Being the largest group of healthcare professionals, nurses play a crucial role in providing direct care for victims in hospitals and responding to individuals’ and communities’ needs, especially during disasters [1]. The nursing faculty should establish a disaster education and training program as a priority and make the necessary preparations to build up nurses’ competence in responding to disasters [1]. Over the past two decades, healthcare organizations and universities have endeavored to develop a specialized educational framework for healthcare professionals’ disaster competencies [1,2,3,4,5,6,7]. For instance, the WHO & ICN (2009) established a framework of disaster nursing competencies with ten domains among the four stages of the disaster management continuum: prevention, preparedness, response, and recovery [1]. Moreover, McCabe and colleagues (2010) developed a public health emergency preparedness framework composed of three essential elements: readiness, willingness, and ability to ensure the probability of quality responses [6].

Research has shown a positive shift in disaster preparedness training programs in the past decade. The significance of disaster education in building participants’ capacity for disaster management has been confirmed [8,9,10,11,12,13,14,15,16]. Other literature examines healthcare professionals’ willingness and ability to report for duty during different disasters or public health emergencies [12,13,17,18,19,20,21,22]. For example, a survey investigated 181 Korean hospital nurses’ competencies in responding to a disaster. Although 82.6% of participants had disaster-related education, 92.3% had no previous disaster experience. A total of 70.2% said they would not know what to do, 60.2% thought they were not well-prepared, and 64.1% believed the workplace should offer adequate support resources [18]. Another survey examined 468 nurses in medical centers in Poland regarding their disaster preparedness and experiences. They believed that triage training is one of the vital predictors of disaster preparedness, yet there was no triage training in their workplace [19]. Most of these nurses had taken basic life support and first aid training, but most had not undergone advanced life support training. Moreover, they desired to receive skills training to handle psychological crises. However, various factors might affect healthcare professionals’ willingness to respond to disasters, e.g., the type of disaster, personal or family safety, education level, self-efficacy, and disaster ability [11,15,17,18,21,23,24]. Many studies have found that interactive, simulation-based, and learner-centered nursing education programs helped prepare healthcare professionals and positively influenced their self-confidence, motivation, and disaster response attitudes [8,11,16,25,26,27,28,29,30].

Recently, Said and Chiang conducted a systematic review of 12 studies involving 1443 nurses from 2001 to 2018, assessing the nurses’ disaster preparedness regarding disaster knowledge, skill competencies, and emotional preparedness [22]. The results found inadequate knowledge regardless of education or training, low skill competencies, and insufficient psychological preparation. However, the study participants had a positive attitude and willingness to care for victims during a disaster. Another updated paper critically analyzed 75 international studies regarding the development of disaster nursing training programs over the past two decades, highlighting the significance and inadequacy of meeting worldwide necessities in disaster education programs [31]. The majority of these studies (70%) were focused on different types of disaster training, especially bioterrorism (13.3%). Although these educational programs adopted various teaching approaches utilizing multiple technologies, the contents mainly emphasized disaster preparedness and response instead of the whole disaster management cycle [31]. The authors recommended organizing undergraduate/postgraduate compulsory disaster nursing education and mandatory hospital in-service training to prepare the nursing staff for responding to mass casualty incidents.

Evidence suggests that disaster preparedness training is vital. Although the *ICN Framework of Disaster Nursing Competencies* was established in 2009 [1] and reviewed in 2018–2019 [32], disaster preparedness training is not an essential component of basic nursing training in Hong Kong [33]. Local studies have recognized that doctors and nurses have low awareness and inadequate preparedness for public health emergencies and disasters [11,34,35,36,37]. Other local evidence has suggested that developing core competency-based training and comprehensive disaster education would benefit healthcare professionals in responding to future public health emergencies and disasters [11,21,36,37,38]. Thus, initiating basic training and enhancing on-the-job training would help improve healthcare professionals’ ability and confidence in the near future.

As future healthcare professionals, nursing students should possess the appropriate knowledge and skills and have a positive attitude when responding to emergency incidents or disasters. Several local academic institutions have introduced disaster education for undergraduate or postgraduate healthcare and nursing students in the past ten years. However, there have been no attempts to examine the effectiveness of these courses or programs. This study is the first to examine the effectiveness of disaster education for undergraduate nursing students in Hong Kong. The study objectives were to improve their:

- knowledge of disaster management;
- willingness to offer help when responding to major incidents or disasters; and
- perceived ability (in terms of confidence) to offer help when responding to major incidents or disasters.

The study offers critical insights into the training’s effectiveness. The results and participants’ feedback could help further develop or refine future disaster education and curricula.

## 2. Materials and Methods

### 2.1. Design

This study adopted a mixed-method design using a single group with pre- and post-intervention comparisons followed by qualitative focus group interviews. The benefits of this two-phased approach provided the research team with the opportunity to review and analyze the survey results in the first phase and then use this information to tailor the guiding questions for the following face-to-face, in-depth focus group interviews. This helped to clarify confusing or significant concerns based on the survey data in this second phase [39]. The mixed-method design allowed participants to express their views and promote exploration approaches. This paper mainly focuses on the first phase’s quantitative results evaluating participants’ knowledge, willingness, and perceived ability when responding to disasters. The qualitative findings of the second phase that revealed the nursing students’ experiences and perceptions of the disaster training course can be found in a recently published paper [11].

### 2.2. Setting and Participants

The study setting was one of the largest self-financed tertiary institutions offering professionally accredited degrees to train nurses and allied health professionals in Hong Kong. The institution offers several undergraduate nursing programs, with more than 1600 general nursing students enrolled during the data collection period. Purposive sampling was adopted. The inclusion criteria were students (a) aged 18 or above, (b) studying nursing programs with previous clinical practice experience, (c) with the ability to understand English and Chinese, and (d) who voluntarily enrolled for the disaster course. The exclusion criteria were students who did not answer the questionnaires completely. For sample size estimation, a power analysis was performed using G*Power 3.10 with power = 0.8, *α* = 0.05 [16], and effect size Cohen’s d = 0.67 [40]. Considering the dropout rate of 16.67–21.30% from previous studies [16,41], the estimated sample size of this study should be more than 25.

### 2.3. Teaching Interventions

We conducted a 45-h disaster preparedness and management course that was based on local and international nursing organizations’ recommendations and findings in the literature. The course contents included theoretical and practical inputs related to the disaster management cycle of prevention, preparedness, response, and recovery phases. It comprised an introduction on the different types and nature of disasters, various stages of the disaster management continuum, local and global disaster management plans, the nurse’s role in responding to disasters and major incidents, emergency life support training, physical and psychological first aid, trauma management skills, etc. The contents were primarily referenced from international and local relevant guidelines and information such as the *ICN Framework of Disaster Nursing Competencies* [1], the Emergency Response System of the Hong Kong Special Administrative Region (HKSAR) [42], the emergency disaster plan of the local Hospital Authority, and international and local trauma-related courses. Trainers of this course have rich clinical and training experience in disaster and emergency nursing. We used various teaching methodologies and action learning activities to stimulate, motivate, and facilitate students’ learning and consolidate what they had learned. The teaching contents included lectures (24 h), problem-based and action learning activities (15 h), disaster and trauma skills demonstrations and practices (4 h), and disaster expert seminar sharing (2 h). Interactive learning activities included crossword puzzles, disaster risk spotting games, disaster management board games, tabletop exercises, computer games, and simulation-based laboratory activities. The simple triage and rapid treatment (START) field triage approach was adopted and taught during the lectures. Tutorial tabletop exercises or a 3D web-based serious computer game further enhanced the students’ understanding of the concept and increased their learning interest. After that, all the participants were allowed to participate in a mass casualty roadway traffic accident simulation-based exercise with student helpers presenting as victims (with trauma makeup).

### 2.4. Outcome Measures

A self-administrated questionnaire comprising four parts was developed. Firstly, the disaster knowledge questions included 15 true or false items based on training contents and relevant disaster information from the World Health Organization [43], for example, “disasters cause deaths at random.” Each correct answer scored one mark, and an incorrect answer scored zero. The total score ranged from zero to 15. A higher total score meant better knowledge. In the second and third parts, one local and five international disaster events were selected to evaluate participants’ willingness and perceived ability to respond to different catastrophic natural and human-caused disasters. The disasters were (1) the 9/11 terrorist attack in the United States, (2) the 512 Sichuan earthquake in China, (3) the 311 Great East Japan Earthquake followed by a tsunami and radiation leakage in Japan, (4) the Lamma Island ferry collision in Hong Kong, (5) the Ebola virus epidemic outbreak in West Africa, and (6) the Formosa Fun Coast water park explosion in Taiwan. A four-point Likert scale ranging from (1) very unlikely to (4) very likely was used. A higher mean score refers to more willingness to offer help in a disaster. A four-point Likert scale ranging from (1) no confidence to (4) very confident in their perceived ability was used. A higher mean score reflects more confidence in their ability to respond to a disaster. The participants were also asked if any significant concerns would influence their willingness and perceived ability as optional supplementary information. These open-ended items aimed to enhance the participants’ substantial concerns and help facilitate in-depth interviews in the second phase. Lastly, personal data were collected, e.g., age, gender, and previous voluntary work experience. Four experienced nurses with 12–25 years of emergency and disaster nursing experience were invited to check the content’s validity. The average scale content validity index (SCVI) was 0.96 once necessary amendments had been made. The measurement reliability was satisfactory with ‘perceived ability’ pre- and post-course α = 0.89 and ‘willingness’ pre- and post-course α = 0.82. Using the questionnaire, a pilot test with 20 nursing students identified any potential problems, time duration, and feasibility. The questionnaire could be completed within 10 min and no adjustments were required.

### 2.5. Ethical Considerations

Before the study’s implementation, ethical approval from the Research Ethics Sub-Committee of the selected institution was sought. The potential participants were well informed about the study’s aims, risks, benefits, procedures, and the voluntary nature of participation with a written information sheet. No personal information could be identified and codes were used to ensure anonymity to replace the participants’ names. Their right to withdraw from the study at any time without any negative consequences affecting their academic performance was emphasized. All the data were kept confidential and encrypted and could only be accessed by the research team. The code list was destroyed after disseminating the research results or kept for seven years after data collection. The electronic database file was deleted from the computer upon the expiration date for the study. Hard copies were shredded, and electronic data were destroyed through deletion.

### 2.6. Data Collection and Analysis

The intervention course was conducted from January 2017 to April 2017. There were 261 potential participants recruited through mass emails and a class announcement before the course commenced. Although this was a compulsory course within the curriculum of the nursing program, the students had their own autonomy to choose to participate in the study or not. Altogether, 180 eligible participants agreed to join with the informed consent signed and pre-intervention questionnaires completed before beginning the course’s first lesson. The post-intervention questionnaires were distributed and collected upon course completion. To avoid participants being identified, they were asked to code themselves with their mother’s initials and the last four digits of their mother’s mobile number in both questionnaires. The executive assistant collected all the completed questionnaires and the research assistant performed the data entry and management. The categorical variables (e.g., age, gender, and previous voluntary work experience) were expressed as frequency and percentage. Paired *t*-tests were used to determine whether there was a statistically significant mean difference between pre-intervention (baseline) and post-intervention scores. Independent sample *t*-tests were adopted to compare the means for two groups of age, gender, and previous voluntary work experience. All analyses were carried out using IBM SPSS Statistics (Version 26.0) (Armonk, NY, USA).

## 3. Results

Overall, 157 of 180 students participated in and completed the pre- and post-intervention questionnaires; 23 were excluded because of incomplete questionnaire responses. Table 1 presents the demographic information of the participants. Most participants were female (128; 81.5%), whereas 29 (18.5%) were male. The majority were aged <23 (*n* = 125, 79.6%), whereas 32 were aged ≥23 (20.4%). More than half of participants (*n* = 82, 52.2%) did not have any voluntary work experience.

As shown in Table 2, positive significant results were found in disaster knowledge (t(156) = −8.12, *p* < 0.01, d = −0.84) and perceived ability (t(156) = −7.95, *p* < 0.01, d = −0.72). Since items in perceived ability and willingness were measured by Likert scales, each of their changes in pre- and post-intervention were further analyzed individually. Perceived ability exhibited a significant increase for each item, with effect sizes ranging from −0.43 to −0.74 (Table 3), which means there was a medium effect size after the intervention. Perceived ability in the face of the six disasters showed statistically significant differences (*p* < 0.01). The highest mean score after the intervention was the Lamma ferry collision (2.63 ± 0.69), followed by the water park explosion (2.46 ± 0.72), the 512 Sichuan earthquake (2.44 ± 0.70), the 9/11 terrorist attacks (2.35 ± 0.72), the 311 Japan earthquake (2.24 ± 0.72), and the Ebola epidemic (2.08 ± 0.82) (Table 3). However, there was no significant improvement in willingness (Table 4).

The outcomes of the intervention were further analyzed by age, gender, and voluntary work experience (Table 5). Most of the subgroups showed improvement after the intervention in disaster knowledge, perceived ability, and willingness. Only a negligible decrease in competence from the baseline in the willingness domain was noted in those participants who were aged below 23, female, and had no previous voluntary work experience. The development of various competencies was equal in different age groups, both sexes, and with or without voluntary work experience, except those aged ≥ 23 who showed a significant increase in mean difference in willingness than those who were below 23.

For the supplementary information regarding the significant concerns that would influence the participants’ willingness to respond to a disaster, the participants’ concerns were summarized and grouped into three primary perspectives that included (1) personal risk perceptions, (2) contextual factors of the disaster events, and (3) organizational support. The participants described concerns about their family members’ safety and their own personal physical and psychological health. Additionally, the nature, types, location, and severity of the disaster events, and the cultural and linguistic differences required for communication in the affected society, were mentioned as factors that may induce hesitation. However, some nursing students emphasized that the employers or the government should provide adequate resource support to respond to disasters, e.g., basic human needs, staffing, personal protective equipment, and material resources. These concerns might be sources of hesitation or reasons for the insignificant improvement in their willingness.

On the other hand, the concerns that would affect the participants’ perceived ability were a lack of confidence in their ability to respond to a disaster regarding (1) the challenges and difficulties that are outside of their expectations, (2) inadequate real disaster exposure or experience, and (3) the nurse’s role in disasters. Some stated that they had increased in terms of their confidence as they had improved disaster knowledge and practical skills training.

## 4. Discussion

Due to the increasing trend, complexity, and severity of different disasters worldwide, adequate and competent staffing is essential for disaster management to ensure the safety and sustainability of care provision. This study reflected a significant improvement in the nursing students’ disaster knowledge and perceived ability to respond to a disaster, although there was no substantial change in willingness. The training course in this study applied various learner-centered interactive activities to facilitate the students’ enjoyment and unite learning with playing [11]. The students could acquire disaster knowledge and hands-on practice skills and experience realistic disaster scenarios. Game-based or game-initiated education for disaster prevention training was recognized as having significant benefits in teaching disaster management and could motivate students’ learning interests [44]. Through tabletop exercises in this study, the students could experience a decision-making process and facilitate their prompt decisions, similar to those that nurses perform in actual situations, in line with previous studies [45]. Moreover, the problem-solving-based field triage simulation exercise in this study could intensify students’ disaster competency and develop students’ critical thinking and clinical decision-making abilities [26,27]. This course comprised problem-based and action learning activities with disaster and trauma skills that aroused the participants’ learning interests. This study enhanced the participants’ knowledge and practical skills that were consistent with action learning that will help improve Hong Kong, Taiwan, Macau, and China nursing students’ capacity to deal with disasters [14].

### 4.1. Knowledge Regarding Disaster Training

Notably, this study demonstrated the effectiveness of the disaster training course in increasing the student participants’ disaster knowledge. Similar to the results of other international studies, there was a positive shift in knowledge [8,9,10,16,25,28]. Some studies have mentioned that disaster preparedness or education knowledge was relatively low or poor at baseline [19,46,47,48]. However, moderate-to-high knowledge levels were found in certain aspects, e.g., triage and basic first aid [48]. Additionally, studies have suggested that more effort could be expended by tertiary nursing institutions’ faculties to further improve or clarify potential misconceptions or myths.

### 4.2. Perceived Ability in Terms of Confidence

For the perceived ability of the study participants, a significant increase was reported for different disaster events with substantial effect sizes. In line with previous literature, disaster training and workplace experience on serious or disaster events would impact personal readiness in responding to disasters [15]. Readiness could be defined as knowing what should be done, having the ability to cope with the situation, and having security and confidence in providing nursing care in responding to disasters [49,50]. In this study, the participants’ results demonstrated significant improvement in their perceived ability to respond to a disaster. However, some of them expressed their concerns in the supplementary information that they did not feel confident due to their roles’ difficulty, unexpected challenges that might happen during disasters, and a lack of disaster management experience. Moreover, results suggest that further training, such as triage or advanced life support training, were required to strengthen their ability and confidence, which echoed previous studies [18,19]. Besides, a local study investigated nursing students’ knowledge and attitudes towards bystander basic life support skills. They found that they had positive attitudes but lacked confidence due to inadequate knowledge and training [51]. The authors suggest that incorporating basic life support training into the college curricula could be a long-term strategy to enhance life support in emergencies that benefits local communities. Additionally, early initiation of disaster training and exposure to certain kinds of major incidents will be more beneficial and prepare them to respond to disasters in their future professional careers.

### 4.3. Willingness to Offer Help

Although there was no significant difference found in this study for the participants’ willingness to respond to different disasters, they showed a positive attitude as evidenced by relatively high mean scores both pre- (mean = 2.98) and post-intervention (mean = 2.96). The participants elaborated their significant concerns regarding willingness through the supplementary information, which included personal risk perceptions, contextual factors of the disaster events, and organizational support. Some nursing students also expressed an increased willingness to join international disaster relief volunteers if the occasion arose. This echoes a previous survey of 484 university nursing students where 77.4% of them declared they would volunteer in a pandemic event if protective garments were supplied [52]. Additionally, 70.7% believed that they had a professional obligation to volunteer during an influenza pandemic.

During the disasters, the provision of care for their own families and the right to self-protection from serious risks were the two significant conflicts causing healthcare professionals’ hesitation. For instance, a local study explored emergency nurses’ experiences after the 2009 H1N1 pandemic [21]. The participants expressed concerns about their physical and psychological wellbeing, as well as their families’ safety. Another survey of 451 Australian emergency nurses’ willingness to work in a disaster found that it was dependent on different factors such as the type of disaster, individual and family concerns, and workplace factors [17]. For instance, some respondents who were living with children were unwilling to report to work. Some respondents would be more willing to report for duty if they possessed relevant disaster knowledge and skills. These studies’ results challenged nurses’ obligation to care for patients in light of personal risks, overwhelming care demands, and limited resources in times of disaster. Indeed, nurses encounter more significant health risks than other healthcare professionals as close patient contact is required for daily routine care and high-risk procedures.

In the past decade, research studies revealed a higher percentage of willingness and ability to report to work during an influenza pandemic, despite the potential risk of infection [21,52,53,54], but a comparatively low percentage of willingness to respond to biological, radiological, chemical, or other human-caused disasters [15,55,56]. In a recent study of 169 Korean nurses, the nurse respondents expressed the highest willingness and perceived disaster competency in a landslide [18]. In contrast, they reported the lowest willingness and competency for human-caused radiative terrorism. In this study, the student participants were more willing to respond to the Lamma Island ferry collision, water park explosion, and 512 Sichuan earthquake that had happened locally in Taiwan and China. The possible explanation is that these disasters occurred in areas with cultural and linguistic similarities for easy communication and accessibility.

According to a recent study by Choi and Lee [18], education level, self-efficacy, and competence in disaster management were the predictors of public health nurses’ willingness to respond to disasters. For those nurses with higher self-efficacy, they were more willing to respond to disasters. Therefore, strengthening nurses’ self-efficacy and improving nurses’ self-confidence through appropriate disaster training might further increase their willingness. In contrast, some recognized their inadequacy in disaster knowledge and competency after learning more about disaster management. In addition, the concerns expressed by the participants regarding their willingness and perceived ability could provide critical insights and further information for educational institutions, healthcare organizations, and disaster relief organizations.

Finally, several significant limitations must be addressed. The study population may not be representative of the general population. All the nursing student participants were recruited from the final year students of a Bachelor of Science in Nursing program in a single tertiary institution. Future studies should consider recruiting a larger sample involving subjects from nursing programs of different educational settings. A recent local study demonstrated the effectiveness of a training program to increase nursing students’ knowledge sustainability, improve their attitude, self-confidence, and intention to help others by conducting a randomized controlled trial with a pre-test, post-test, and six-month follow-up research design [57]. Thus, a similar research design is recommended for future studies instead of a single group with a pre- vs. post-intervention research design.

## 5. Conclusions

This study evaluated the effectiveness of disaster education for undergraduate nursing students’ knowledge, willingness, and perceived ability. The participants demonstrated significant improvement in disaster knowledge and perceived ability but no substantial change in willingness to respond to disasters. The results showed that their reluctance or hesitation in willingness was mainly related to their concerns regarding personal disaster risk perceptions on health issues, contextual factors related to various disasters, and organizational support from their employers or the government. The study results reflected that an appropriate course design with interactive, simulation-based, and learner-centered nursing education programs could positively influence participants’ knowledge and self-confidence, and that disaster education of this kind could be promoted to prepare future nursing professionals. The contents should further be enhanced to include elements regarding the students’ willingness. However, local government, healthcare organizations, and tertiary institutions should provide appropriate strategies, adequate resource support, and proper training to alleviate the nursing students’ and professionals’ concerns and to enhance their willingness to respond to disasters. The evidence from this study suggests that incorporating disaster training into tertiary education curricula as an essential component of basic training for nursing professionals could be a long-term strategy to expand the competent workforce and prepare nursing professionals for future disasters. Furthermore, introducing disaster education as part of extracurricular activities or elective subjects for other healthcare or general students of tertiary institutions could be another beneficial strategy for preparing a more competent workforce for future public health emergencies or disasters.

## Figures and Tables

**Table 1:** Demographic information of participants (Total = 157).

	Frequency	Percentage
**Age**		
<23	125	79.6
≥23	32	20.4
**Gender**		
male	29	18.5
female	128	81.5
**Previous voluntary work experience**		
yes	75	47.8
no	82	52.2

**Table 2:** Comparison between pre- and post-intervention for various measurements.

	Pre-Intervention Mean (SD)	Post-Intervention Mean (SD)	Mean Difference	t	df	Cohen’s *d*
Disaster knowledge	6.29 (1.94)	8.16 (2.53)	−1.87	−8.12	156	−0.84 **
Willingness	2.98 (0.50)	2.97 (0.50)	0.01	0.24	156	0.02
Perceived ability	1.93 (0.61)	2.36 (0.59)	−0.43	−7.95	156	−0.72 **

*p* < 0.01 **.

**Table 3:** Comparison between pre- and post-intervention for perceived ability.

	Pre-Intervention Mean (SD)	Post-Intervention Mean (SD)	Mean Difference	t	df	Cohen’s *d*
1. 911 terrorist attack	1.82 (0.72)	2.35 (0.72)	−0.53	−7.69	155	−0.74 **
2. 512 Sichuan earthquake	2.01 (0.75)	2.44 (0.70)	−0.43	−6.33	155	−0.59 **
3. 311 Japan earthquake	1.81 (0.69)	2.24 (0.72)	−0.43	−6.09	155	−0.61 **
4. Lamma ferry collision	2.30 (0.86)	2.63 (0.69)	−0.33	−4.66	154	−0.43 **
5. Ebola epidemic	1.57 (0.66)	2.08 (0.82)	−0.51	−6.96	156	−0.68 **
6. Water park explosion	2.05 (0.78)	2.46 (0.72)	−0.41	−6.36	155	−0.55 **

*p* < 0.01 **.

**Table 4:** Comparison between pre- and post-intervention for willingness.

	Pre-Intervention Mean (SD)	Post-Intervention Mean (SD)	Mean Difference	t	df	Cohen’s *d*
1. 911 terrorist attack	2.98 (0.71)	2.95 (0.68)	0.03	0.48	156	0.04
2. 512 Sichuan earthquake	3.07 (0.69)	3.05 (0.65)	0.02	0.33	155	0.03
3. 311 Japan earthquake	2.87 (0.81)	2.90 (0.77)	−0.03	−0.40	156	−0.04
4. Lamma ferry collision	3.37 (0.63)	3.27 (0.70)	0.10	1.38	156	0.15
5. Ebola epidemic	2.54 (0.80)	2.57 (0.83)	−0.03	−0.43	156	−0.04
6. Water park explosion	3.03 (0.64)	3.04 (0.65)	−0.01	−0.20	156	−0.02

**Table 5:** Outcome of intervention by age, gender, and voluntary work experience.

Outcome	Age		*p* Value *	Gender		*p* Value *	Voluntary Work Experience		*p* Value *
	<23 (*n* = 125)	≥23 (*n* = 32)		Male (*n* = 29)	Female (*n* = 128)		Yes (*n* = 75)	No (*n* = 82)	
**Mean (SD) knowledge score**									
At baseline	6.32 (1.98)	6.19 (1.82)	0.73	6.10 (1.86)	6.34 (1.97)	0.56	6.24 (1.78)	6.34 (2.09)	0.75
After intervention	8.02 (2.46)	8.69 (2.76)	0.19	8.45 (2.78)	8.09 (2.48)	0.50	8.56 (2.62)	7.79 (2.41)	0.06
Mean change (SD) from baseline	1.70 (2.82)	2.5 (3.08)	0.16	2.34 (3.05)	1.76 (2.84)	0.32	2.32 (3.00)	1.45 (2.72)	0.06
**Mean (SD) willingness score**									
At baseline	2.97(0.52)	3.02 (0.45)	0.58	2.98 (0.46)	2.98 (0.51)	0.94	3.02 (0.46)	2.94 (0.54)	0.33
After intervention	2.90 (0.49)	3.21 (0.48)	0.00	3.11(0.51)	2.93 (0.49)	0.08	3.05 (0.48)	2.89 (0.50)	0.04
Mean change (SD) from baseline	−0.06 (0.57)	0.19 (0.55)	0.03	0.13 (0.55)	−0.04 (0.57)	0.14	0.04 (0.55)	−0.05 (0.59)	0.34
**Mean (SD) perceived ability score**									
At baseline	1.89 (0.63)	2.06 (0.50)	0.16	2.28 (0.46)	1.85(0.61)	0.00	1.94 (0.58)	1.92 (0.64)	0.83
After intervention	2.34 (0.60)	2.42 (0.56)	0.51	2.61 (0.53)	2.30 (0.59)	0.01	2.42 (0.64)	2.30 (0.54)	0.19
Mean change (SD) from baseline	0.45 (0.67)	0.36 (0.74)	0.50	0.33 (0.59)	0.45 (0.70)	0.39	0.49 (0.72)	0.38 (0.65)	0.34

* Independent-sample *t*-tests.

## Data Availability

The data presented in this study are available on reasonable request from the corresponding author.
